# The use of maize haploidy inducers as a tool in agricultural plant biotechnology

**DOI:** 10.18699/VJGB-22-85

**Published:** 2022-11

**Authors:** A.V. Ulyanov, A.V. Karlov, E.B. Khatefov

**Affiliations:** Federal Research Center the N.I. Vavilov All-Russian Institute of Plant Genetic Resources (VIR), St. Petersburg, Russia; Saratov State University, Saratov, Russia; Federal Research Center the N.I. Vavilov All-Russian Institute of Plant Genetic Resources (VIR), St. Petersburg, Russia

**Keywords:** maize, haploidy, haploid inducer, haploid, doubled haploid, tetraploid, rediploid, кукуруза, гаплоидия, гаплоиндуктор, гаплоид, дигаплоид, тетраплоид, редиплоид

## Abstract

The discovery of the ability of some mutations to stimulate haploidy during hybridization made it possible to create one of the most promising and sought-after trends in the f ield of reproductive biology. Haploid inducers created on their basis are capable of increasing the frequency of haploidy up to 15 %. The improvement of the existing haploid inducer lines and the search for new genes that contribute to a high frequency of haploidy are underway. Along with these studies, the f ield of application of haploid inducers in genetics and plant breeding is expanding. Haploid inducers carrying R1-nj genes for anthocyanin pigmentation of the seed and embryo are able not only to mark the hybrid embryo and identify haploid genotypes, but also to detect genes that suppress the anthocyanin color of the grain, like C1-I, C2-Idf, and In1-D. Depending on their quantity, the phenotypic manifestation of the gene in the seed varies. Haploidy is widely used for accelerating hybrid breeding and obtaining both new maize lines with improved traits and their sterile counterparts. By introducing certain genes into the genome of the improved line, breeders can use the doubled haploid (DH) breeding technology to accelerate the creation of pure lines carrying the desired gene. Haploid inducer maize lines and their tetraploid analogs are used in the selection of rediploid maize lines by their resynthesis from tetraploid genotypes. In 2019, Syngenta Company synthesized a haploid inducer maize line carrying a CRISPR/cas construct capable of simultaneously stimulating haploidy and editing the genome at a specif ied DNA site. Thanks to this technology, it became possible to improve haploid inducers by introducing various CRISPR/cas constructs into the haploid inducer genome for editing any DNA site. Maize haploid inducers are widely used in doubled haploid wheat breeding. The f irst experiments showed that the most effective haploid inducer for stimulating haploidy in wheat is maize pollen. Researchers are intensively searching for other ways of using maize haploid inducers in plant breeding.

## Abstract (ru)

Использование гаплоиндукторов в гибридной селекции растений является одним из перспективных и востребованных направлений в области репродуктивной биологии. Продолжается совершенствование уже существующих линий-гаплоиндукторов и поиск новых генов, способствующих повышению частоты гаплоидии. Наравне с этими исследованиями расширяется область применения гаплоиндукторов в генетике и селекции растений. Гаплоиндукторы, несущие гены R1-nj, которые маркируют антоциановую окраску верхушки зерновки и зародыш, используются не только для выявления гибридных зародышей (окрашенный зародыш) и гаплоидных генотипов (неокрашенный зародыш), но и для обнаружения генов, репрессирующих антоциановую окраску зерна, таких как как C1-I, C2-Idf, In1-D. В зависимости от количества генов изменяется их фенотипическое проявление в зерновке. Гаплоидия широко применяется для ускорения гибридной селекции и получения новых линий кукурузы с улучшенными признаками и их стерильных аналогов. Вводя те или иные гены в геном улучшаемой линии, селекционеры могут ускорить создание чистых линий, несущих нужный ген, методом дигаплоидной (DH) селекции. Гаплоиндукторные линии кукурузы и их тетраплоидные аналоги используются в селекции редиплоидных линий кукурузы методом ресинтеза из тетраплоидных генотипов. Фирма Syngenta в 2019 г. синтезировала гаплоиндукторную линию кукурузы, несущую в спермиях пыльцевого зерна конструкцию CRISPR/cas, которая способна к одновременному стимулированию гаплоидии и редактированию генома на заданном участке ДНК. Благодаря этой технологии стало возможным совершенствование линий гаплоиндукторов кукурузы с помощью введения различных конструкций CRISPR/cas в ее геном для редактирования на любом участке ДНК. Гаплоиндукторы кукурузы широко применяются в селекции дигаплоидной пшеницы. Первые опыты показали, что наиболее эффективным гаплоиндуктором для стимулирования гаплоидии на пшенице является пыльца кукурузы. Исследователи ведут интенсивный поиск других возможностей использования гаплоиндукторов кукурузы в селекции растений. В данном обзоре рассмотрено современное состояние в технологии гаплоиндукции у растений.

## Introduction

The creation of inbred lines is the main goal of hybrid maize breeding. As a result of long-term self-pollination, breeders obtain homozygous lines for using them as parents in hybrid combinations. This part of the breeding work is the longest and most labor-consuming. Doubled haploid (DH) maize lines are usually created in vivo by crossing with haploid inducers that are able to stimulate the formation of haploid embryos in a fraction of the seeds of the mother plant, which serves as a donor of genetic material for the future doubled haploid line. The obtained haploid plants are subjected to the chromosome number doubling to diploid, as a result of which homozygous doubled haploid seeds are formed in the cob. This method made it possible to quickly obtain completely homozygous lines, which, in turn, significantly reduced the time and means spent by breeders on creating a hybrid combination.

The existing haploid inducer lines are characterized by a low frequency of haploid induction and require additional research to improve this trait. The research on improving the frequency of haploid induction in haploid inducer lines has caused interest in the mechanism of this phenomenon and the study of its genetics. The area of haploid inducers and haploidy application has stepped far beyond the limits of only obtaining doubled haploid lines; new technologies in genetics and plant breeding are being elaborated on this basis.

The present paper discusses various applications of maize haploid inducers and haploid induction, which are important for research in the field of basic and applied genetics of maize and other crops. Also, the paper explains their genetic basis, lists known genes and quantitative traits loci, and discusses different approaches to breeding for developing haploid inducer application technologies.

## Progress in the maize haploid inducer breeding

The rapid development of hybrid maize seed production since the middle of the 20th century has contributed to the development of breeding inbred lines with the maximum genome homozygosity. An effective method for improving the quality of seed production and for the accelerated production of inbred lines, as well as for breeding for economically valuable traits, to this day is, and remains, the use of haploid induction for the creation of doubled haploid lines. For a long time, inbred breeding has been the most common method of obtaining inbred lines. This method had many disadvantages, one of which is the impossibility of achieving complete homozygosity of all allele loci in an inbred line even after 10 inbreeding rounds. A high degree of homozygosity is necessary to achieve maximum uniformity of all traits of importance for breeding (uniformity of emergence, dates of flowering and ripening, of grain quality, etc.) and improvement to perfection of the seed production quality, the manufacturability of hybrid combinations obtained on its basis and, accordingly, improvement of the commercial products quality. A breeder spends 10 years to create lines with a fraction of homozygotes equal to 99.90 %, and up to 14 years of breeding work (inbreeding) to achieve the fraction of 99.99 %. For the resulting heterotic hybrids, it is very important to achieve a high degree of homozygosity, since it determines the uniformity of phenotypic traits, the synchronism in the onset of phenophases in plant development and maturation, which, in turn, affects their manufacturability in seed production and industrial production of marketable products.

The introduction of the first haploid inducers into breeding practice made it possible to obtain inbred maize lines with complete homozygosity for all gene loci in a short time. Thanks to making hybrid breeding a common breeding practice, the United States quickly occupied a leading position in the world in the production of hybrid maize in the middle of the 20th century. In addition to improving seed quality in seed production, haploid inducer breeding is widely used for creating inbred maize lines. The rapid production of pure doubled haploid maize lines within 2–3 years allows breeders to qualitatively evaluate more lines and genotypes in a shorter period of time than the previously used inbreeding method (Seitz, 2005; Ravi et al., 2011). Along with that, the possibilities of selecting valuable gene alleles and their rapid introduction into the genome of inbred genotypes have been broadened. The use of doubled haploid breeding technology allows breeders to use haploidy inducer (HI) lines each year to produce millions of recombinant plants based on DH lines derived from F1 or F2 generations. This makes it possible to avoid six or more generations of self-pollination, which are usually required to obtain inbred lines used not only in hybrid breeding, but also for genetic mapping, identification of trait introgression, and genetic studies of quantitative traits (Chang, Coe, 2009). In addition, the technology for obtaining DH lines broadens the possibilities for both improving the quality of selection during breeding and for more accurate phenotyping at different stages of inbreeding (Yan et al., 2017).

Haploid induction methods are widely used in many areas of breeding not only maize, but also other crops. The phenomenon of haploidy has been studied insufficiently and raises many questions, which researchers have to answer – from understanding the mechanism of haploidy to exploring all the possibilities of its use in genetic and breeding research.

The first description of haploidy was made by Dorothy Bergner in 1922 on Jimson weed (Datura stramonium) (Blakeslee et al., 1922). Some time later, haploid genotypes were found in wheat (Gains, Aase, 1926) and tobacco (Clausen, Mann, 1924). Still later, haploidy was described in all major cultivated plants, i. e. wheat, rye, maize, rice, barley, sorghum, potato, tobacco, cotton, flax, beet, cabbage, cucurbits, cucumber, tomato, as well as in such forage grasses as bluegrass, brome grass, timothy, alfalfa, vetch, etc. (Kirillova, 1966). Haploids were also found in many other plants, but the frequency of haploidy remained insignificant (0.001–0.01 %) for its use in breeding

Haploids can be produced experimentally by disrupting androgenesis or gynogenesis, the fertilization mechanisms of flowering plants (Asadova et al., 2020). The progeny obtained via androgenesis is characterized by haploid or doubled haploid germplasm and inherits only paternal traits (Astaurov, 1977; Darevsky et al., 1985; Golubovsky, 2000). An organism obtained by means of gynogenesis carries the haploid or doubled haploid material of the mother plant and inherits only the characteristics of the mother form (Astaurov, 1966; Strunnikov, 1998). An organism formed by apomixis carries the haploid or doubled haploid material of the mother plant and inherits only characteristics of the mother form (Takhtadzhyan, 1980; Batygina, 2000).

Serious progress in haploid induction technology was made only 40 years after the discovery of haploidy in plants by Guha and Maheshwari, who obtained haploids of Jimson weed (Datura stramonium) in vitro through anther culture (Guha, Maheshwari, 1964). A real breakthrough in the breeding of hybrids and their parental lines was made thanks to the creation of the first haploid inducers and doubled haploid maize lines, which were first obtained by S. Chase (Chase, 1949). In his studies, he established that the occurrence of haploid plants in crops of commercial inbred maize lines has a frequency of 0.1 % (Chase, 1947). He supposed that diploid pure lines can be obtained with the help of haploids; this suggestion could not but attract interest of breeders and geneticists.

When Chase published the results of his research, work began around the world on the search for maize lines capable of stimulating haploidy with a certain frequency and the development of haploid inducers with anthocyanin marker genes on their basis. The first haploid inducer of this kind, Stock 6, was obtained in 1959 by Ed Coe, and it had a haploid induction frequency of 2–3 %, which at that time was a sufficiently high value to start its introduction into breeding practice and for its commercial use in hybrid maize breeding (Coe, 1959). Subsequently, breeders managed to increase the haploidy frequency up to 7–15 % in newly created haploid inducers derived from Stock 6 by various breeding methods (Eder, Chalyk, 2002; Xu et al., 2013; Asadova et al., 2020). The first studies on maize haploid induction were started in the USSR by V.S. Tyrnov and A.N. Zavalishina at the Saratov University under the guidance of Prof. S.S. Khokhlov (Khokhlov et al., 1976; Tyrnov, Zavalishina, 1984). They created the first domestic haploid inducer maize lines based on the AT-1 line, which served as a source for the creation of most domestic haploid inducers. The number of effective haploid inducers used in the world for producing maize haploids varies within 50–60 lines, most of which were created on the basis of Stock 6 (Hu et al., 2016).

## Physiological mechanisms of haploid induction in maize

Much research has been devoted to exploring the mechanism of haploidy manifestation in plants, which showed that it is based on the phenomenon of parthenogenesis (Shishkinskaya, Yudakova, 2009; Yudakova, 2017), or gynogenesis, when seeds form an embryo that develops from an unfertilized egg, with a triploid endosperm, resulting from the fusion of the sperm with the central cell of the embryo sac (Takhtadzhyan, 1980; Batygina, 2000). The method of obtaining haploids based on the in vitro gynogenesis is used in the breeding of onion (Allium cepa L.) (Campion et al., 1992), sugar beet (Beta vulgaris L.) (Doctrinal et al., 1989), and some trees in vivo (Borodina, 1982). In onions (A. cepa), gynogenesis occurs in the culture of flower buds or ovaries (Keller, 1990). It should be noted that the temperature regime in which the donor plant grows before flowering, plays a decisive role for the induction of gynogenesis (Michalik et al., 2000).

Haploidy in maize under natural conditions can result from the disruption of one of the two sperms fusion with the egg, or their elimination after penetration into the embryo sac during fertilization (Tyrnov, Khokhlov, 1974, 1976). The mutations accumulated in the genome, which disrupt the fertilization process in maize, contribute to an increase in the frequency of haploidy both in own and in hybrid cobs during cross-pollination. A search for such mutations and identification of their sources are essential for creating haploid inducer maize lines (Khokhlov et al., 1976). It has been established that haploid embryos can form during distant hybridization (Kasha, Kao, 1970; Laurie, 1990).

The mechanisms of haploids appearance during fertilization of the embryo sac in flowering plants are still being elucidated, nevertheless in most cases, normal double fertilization occurs with the formation of a zygote and endosperm, but several subsequent mitotic divisions lead to the elimination of paternal chromosomes, both in the zygote and endosperm cells (Chaikam et al., 2019). To explain this process in the haploid inducer lines obtained from Stock 6, two hypotheses have been proposed, namely the single fertilization hypothesis and the chromosome exclusion hypothesis. The former was put forward first (Sprague, 1929, 1932). According to this hypothesis, one of the sperms cannot fuse with the egg, which triggers the development of the haploid embryo. Bylich and Chalyk found that about 6 % of the pollen in the haploid inducer ZMS (Zarodyshevy [Embryo] Marker Saratovskiy) line had sperms of different size. The scientists supposed that one of the sperms successfully fertilized the endosperm or egg, while the other was incapable of fertilization due to its abnormality (Bylich, Chalyk, 1996). B. Chen et al. found that poorly developed eggs of immature embryo sacs do not develop a well-defined embryo despite normal endosperm formation, which suggests a single fertilization by only one of the two sperms (Chen B. et al., 2016; Tian et al., 2018).

According to the second hypothesis, which adheres to the theory of chromosome elimination, double fertilization occurs normally, but the haploid inducer chromosomes are eliminated during several zygotic divisions. This hypothesis is corroborated by several facts, one of which is the appearance of several micronuclei in some ovules that were fertilized with haploid inducer pollen followed by elimination of haploid inducer chromosomes (Wedzony et al., 2002; Fischer, 2004; Li L. et al., 2009). Later, X. Li et al. showed that chromosome elimination is directly related to the induction of the haploid genotype in the embryo, and the elimination process occurs gradually, so they proposed two models for the mechanism of haploid induction in maize according to the importance of chromosome elimination in both sperms (Li X. et al., 2017). Model I suggests the fusion of normal or slightly defective sperms with normal eggs to form diploid hybrid kernels in the cob. According to Model II, haploids can be formed only when the sperm fertilizes the egg and undergoes further elimination of chromosomes, while fertilization of the central cell by the same sperm leads to formation of abortive seeds.

## Genetic mechanisms of haploid induction in maize

An important step in the study of haploid induction was the determination of the localization of a group of genes promoting haploidy, which was carried out on the Stock 6 haploid inducer line. It was found that the most frequently used genes in modern maize haploid inducers are the ones located in the qhir1, qhir11, and qhir12 regions of chromosome 1 (Hu et al., 2016). However, an assessment of the haploid induction frequency in genotypes containing the qhir11 and qhir12 subregions showed that only qhir11 has a significant effect on haploid induction (Nair et al., 2017). In the qhir11 region, the gene encoding phospholipase A has been identified as responsible for haploid induction by three independent research groups and named MATRILINEAL (MTL) (Kelliher et al., 2017), NOT LIKE DAD (NLD) (Gilles et al., 2017) and ZmPLA1 (Liu C. et al., 2017). It was shown that the mutant gene MTL/NLD/ZmPLA1 has a 4 bp insert in exon 4 (CGAG), which, in turn, has a haploid induction effect. It turned out that this insert shifts the reading frame, thereby changing the sequence of 20 nucleotides and forming a premature stop codon, which shortens the protein by 29 amino acids in comparison with the protein derived from the wild-type gene. This insert was present in all maize haploid inducer lines that originated from Stock 6 (Liu C. et al., 2017). It should be noted that this mutation was not found in teosinte, the ancestral form of maize, which suggests that this mutation appeared after maize domestication (Liu C. et al., 2017).

Another equally interesting discovery is that some gene editing and knockdown events increase the frequency of haploidy, thus indicating the possibility of creating an inducer with a higher haploid induction frequency by modifying the MTL gene. This gene encoding patatin-like phospholipase is expressed in maize pollen (Gilles et al., 2017; Kelliher et al., 2017; Liu C. et al., 2017). In turn, the altered phospholipase affects both the rate of pollen germination on maize stigmas and the growth of pollen tubes to the embryo sacs, significantly slowing down their germination (Kim et al., 2011), which explains the haploid inductive ability of maize (Xu et al., 2013; Kelliher et al., 2017). However, the mechanisms of haploidy based on single fertilization (Sarkar, Coe, 1966) or postzygotic genome elimination (Qiu et al., 2014; Li X. et al., 2017) still leave many questions unanswered and remain unclear.

Recent studies by Chinese scientists of the factors affecting the frequency of haploidy have characterized the membrane protein DUF679 (ZmDMP) in a line that is not related to the Stock 6 haploid inducer (Zhong et al., 2019). The researchers established that this gene is localized on chromosome 9 in the qhir8 region. It was proven that a mutation in this gene in combination with a mutant haploid induction MTL/ZmPLA1/NLD gene (containing a 4 bp insert) significantly enhances haploid induction. At the same time, the authors noted that the ZmDMP gene mutation itself without interaction with mlt/pla1/nld does not cause an increase in the frequency of haploid induction. A wildtype ZmDMP knockout resulted in a haploid induction frequency of 0.1–0.3 % in the absence of a mutation in the MTL gene, while in combination with such a mutation it led to a 5–6-fold increase in the frequency of haploid induction. The results show that a mutation in the MTL gene is critical for the high frequency of haploids induction due to the effect of ZmDMP. The mechanism by which the MTL gene determines haploid induction and how the interaction of this gene with ZmDMP increases the haploid induction frequency remains to be elucidated.

## The use of maize haploid inducers for marking haploid seeds in genotypes with colored and non-colored seeds

In 1949, S. Chase (Chase, 1949) proposed to introduce genetic markers for haploid inducer maize lines, the meaning of which was in the possibility to observe the phenotypic manifestation of dominant traits in hybrid offspring from such crosses, and of recessive ones in matroclinic haploids (Gutorova et al., 2016). The use of dominant markers in haploid inducer maize lines greatly simplifies the work of culling hybrid diploid seeds and improves the quality of selection of haploid ones, which lack these markers. Thanks to the haploid inducers labeling method proposed by Chase, a variety of created haploid inducers has significantly increased the effectiveness of this technology (Nanda, Chase, 1966; Greenblatt, Bock, 1967; Chase, 1969).

Most of the phenotypic markers used to distinguish haploid seeds from diploid ones are represented by dominant genes with the effects of anthocyanin (purple) markers on various parts of the seed and plant. In the most popular haploid inducer maize lines, the visual differentiation of haploid and diploid seeds is based on the marker of purple color of the embryo, which is encoded by the R1 – navajo gene (R1-nj), and the absence of a dominant allele blocks the synthesis of anthocyanins in the aleurone layer of the seed (Ford, 2000). It should be noted that the expression of the R1-nj anthocyanin marker in hybrid maize seeds can vary significantly depending on the genotype of the maternal line used in the hybrid combination, the genotype of the haploid inducer itself, and environmental factors (Chase, 1952; Röber et al., 2005; Kebede et al., 2011; Prigge et al., 2011).

For a more reliable selection of haploid genotypes, other genes controlling anthocyanin biosynthesis (purple color) were used in addition to the R1-nj gene; these are A1, A2, C2, Bz1, Bz2, and another regulatory gene C1, the expression of which was based on the inhibition of anthocyanin synthesis in homozygotes of recessive alleles of any of these genes, i. e. of A1, A2, C2, Bz1, or Bz2 (Chase, Nanda, 1965; Geiger, Gordillo, 2009). In addition, the dominant anthocyanin synthesis inhibiting genes C1-I, C2-Idf, and In1-ID can influence the purple seed color phenotype (Coe, 1994; Eder, Chalyk, 2002). The studies conducted at CIMMYT suggested that the expression of the purple seed pigmentation by the R1-nj gene is inhibited in 8 % of crosses of haploid inducers with various maize genotypes (Röber et al., 2005; Prasanna et al., 2012). The Pl1 (Purple1) gene causes the light-dependent production of anthocyanins in roots. This effect provides an additional way to distinguishing between haploid and diploid genotypes. If the anthocyanin synthesis inhibitor genes (C1-I, C2-Idf, and In1-ID) caused the incorrect classification of seeds in the R1-nj marker, the effect of the Pl1 gene makes it possible to distinguish hybrid seeds by the presence of purple pigmentation in the primary (embryo) roots of the putative haploid maize seeds. However, the roots of seedlings of some genotypes can turn purple when exposed to light, which in such a case makes the classification based on Pl1 erroneous. A combination of Pl1 with the B1 (Booster1) genes leads to the production of anthocyanins in the coleoptile of the seedling, leaf tip, and primary root. Plants homozygous for alleles of the B1 and Pl1 genes develop a dark purple color on the cob and stem envelope (Coe, 1994), but these markers are effective for genotypes that do not contain pigments in the aleurone layer or various parts of the recipient maize plant.

With the advent of the possibility of analyzing the chemical composition of grain by IR spectrometry, the possibilities of labeling haploids have expanded. In 2007, V. Rotarenco suggested using the oil content in the seed embryo as a marker of haploid seeds (Rotarenco et al., 2007, 2010). The analysis of the chemical composition of the embryo by NMR showed significant differences in the oil content in diploid and haploid seeds, which amounted to 5.26 and 3.42 %, respectively (Chen S., Song, 2003). Based on these results, L. Li et al. crossed a high-oil line with the Stock 6 line and used the resulting hybrid for creating the haploid inducer CAUHOI with a haploid induction frequency of up to 2 %, and an oil content in the seed of up to 7.8 % (Li L. et al., 2009). This haploid inducer makes it possible to significantly broaden the use of haploid inducer lines for obtaining haploids of various maize genotypes, characterized by the presence of purple color in various parts of the plant and seed, or inhibitors of the phenotypic manifestation of the R1-nj genes. Studies by Z. Liu et al. (2016) proved that the oil content in the seed embryo makes it possible to identify haploid genotypes with an accuracy of at least 90 % (Liu Z. et al., 2016).

## The use of maize haploid inducers for the resynthesis of maize polyploid genotypes

The creation of effective haploid inducers made it possible to shape a new direction in breeding, i. e. the creation of rediploid maize using the resynthesis of polyploid genotypes. This method was first proposed for maize by Shatskaya and Khatefov in 2009. The authors of this method proposed two ways for breeding rediploid maize lines (Khatefov, Shatskaya, 2009). The first option suggested resynthesis from hybrid triploid seeds without the use of a haploid inducer. It was a very cumbersome and laborious technique, which did not find wide application in breeding. The second method proposed the use of diploid and tetraploid analogs of haploid inducer lines in a scheme of crossing with tetraploid sources. This method was much simpler and more convenient to use, provided that a breeder had a tetraploid analog of the haploid inducer line. The use of the method of rediploidization of tetraploid genotypes made it possible to create a series of rediploid maize lines from the tetraploid synthetic population MRPP20.

The further testing of the breeding value of the collection of rediploid lines obtained by the resynthesis from tetraploid populations showed their high effectiveness in hybrid maize breeding (Khatefov, Matveeva, 2018; Khatefov et al., 2019, 2021). The use of this method for the resynthesis of natural tetraploid genotypes and populations of cultivated and wild maize relatives opens up great prospects for broadening the genetic polymorphism of the initial breeding material and maize breeding improvement.

## The use of maize haploid inducers for creating sterile analogs of CMS maintainer lines

Along with the spread of the method for creating doubled haploid lines for hybrid maize breeding, the problem of creating their sterile analogs has appeared over time. This is a complex problem to solve, because a sterile analogue is created from the sterile (S) cytoplasm, which is the maternal form in the hybrid maize breeding. It is obtained through a series of backcrosses of the nuclear material source of normal (N) cytoplasm from a maintainer line serving as the paternal form. In this breeding scheme, it becomes impossible to use a haploid inducer line to obtain a sterile analog by the traditional way, when the haploid inducer serves as the paternal form, since the doubled haploid analog obtained from sterile cytoplasm will be sterile and impossible to propagate after doubled haploidization.

A method for creating sterile analogs of maize lines through androgenesis using CMS-ig haploid inducers carrying the ig1 mutation was found at the P.P. Lukyanenko National Grain Center (Kermicle, 1969, 1971, 1994). The method is based on the principle according to which the sperm nucleus replaces the egg nucleus. The proposed method stipulates the fusion of one of the sperms from the source of the rfrf genes with the egg of the haploid inducer line, which serves as the maternal form created on the basis of M- and S-type CMS. Similar to the haploid inducer lines used as a source of pollen, the haploid inducer maternal lines carry alleles of the ig1 genes in combination with the R1- nj, Pl1, and B1 genes. Maternal forms of haploid inducers, homozygous for the ig1 mutation, exhibit a long phase of nuclear-free divisions, which ends with the formation of nuclear- free eggs (Evans, 2007). When hybridizing the paternal form, which carries homozygotes of alleles of the rfrf nuclear genes (maintainer) and a source of sterile cytoplasm (maternal form), a sterile analog of the paternal line with complete sterility is formed in seeds which set in the cobs of the maternal haploid inducer line (Chumak, 1977; Shatskaya, Shcherbak, 1999). To multiply the sterile analogue in future, only the paternal line, which served as a source of pollen for haploid induction, is used as a pollinator. Thanks to this method, the Maize Department of the P.P. Lukyanenko National Grain Center has been creating sterile analogs of maize lines for two years, it being much more efficient than the traditional method of saturating crosses, which takes 8 or more years of breeding work.

## CENH3-mediated haploid induction

In 2010, Ravi and Chan reported that a CENH3 mutant gene can induce haploidy in crosses with wild-type Arabidopsis thaliana (Ravi, Chan, 2010). This CENH3 mutant was created by replacing the endogenous N-terminal (N-ter) tail of CENH3 with a fluorescent GFP protein fused with the N- ter tail of a normal histone 3.3, which partially complements the lethal phenotype of the cenh3 null mutant. Pollination of this GFP-tailswap transgenic line with the wild-type pollen resulted in the induction of ~30 % haploid embryos with only male (wild-type) genome. GFP-tailswap-tagged female chromosomes are lost during early zygote divisions, creating a high frequency of paternal haploid embryos along with aneuploid (~30 %) and normal diploid embryos. In the future, this scheme of haploid induction can be applied to other important agricultural crops (Tek et al., 2015).

Attempts to edit the N-terminal (N-ter) tail of CENH3 have been successful with maize (Tek et al., 2015), tomato, and rice (Kalinowska et al., 2019). Although the rate of natural haploid induction was relatively low (0.065–0.86 % in maize, 0.2–2.3 % in tomato, and 0.3–1.0 % in rice), these experiments demonstrated the feasibility of haploid induction by changing the N-terminal tail of CENH3 in cultivated monocots. Sanei et al. (2011) found that CENH3 of H. bulbosum is inactive and/or CENH3 of H. vulgare could not be introduced into the paternal form in a classical barley cross (H. vulgare × H. bulbosum) because it leads to the formation of haploids with the maternal genome only. Studies by Zhao et al. (Zhao et al., 2013) reported that CENH3 does not directly contribute to the ability to induce haploids. This finding is consistent with the QTL mapping analysis (Prigge, Melchinger, 2012). However, the authors suggest that CENH3 may influence the elimination of chromosomes of the haploid inducer line during the formation of haploids in maize. The authors did not find any differences in the coding sequence and the level of mRNA expression between the inducer and non-inducer lines, while the regulatory levels of CENH3 (splice variants, translation, modification, etc.) may differ. The results of the study showed that CENH3 acts epigenetically in plants (Han et al., 2009; Ravi, Chan, 2010; Sanei et al., 2011). This new knowledge opens up broad prospects for researchers involved in the development of haploid inducer lines for agricultural crops for which it is difficult to create haploid inducers using classical breeding methods.

## The use of the CRISPR/cas system for generating edited dihaploid lines

Over the past decade, the use of the CRISPR/cas method in agriculture has expanded significantly. To obtain pure lines with an edited genome, various haploid inducers, including those of maize, are widely used, which allows scientists to accelerate the breeding process and create plants with the desired traits in a short time. In 2019, two groups of scientists from America and China led by Kelliher and Wang, respectively, independently of each other decided to combine the CRISPR/cas editing method and in planta haploid induction. They used two HI-Edit haploid inducers (Kelliher et al., 2019) and IMGE haploid inducer (Wang et al., 2019). They conducted studies on their transformation with Agrobacter carrying a vector that contained the CRISPR/cas sequence. The transformed haploid inducer lines were crossed with elite maize lines. When the hybrid seeds matured, the isolated haploid seeds were tested for the presence of mutations (transformation event markers) in the aimed (target) region of the gene. The studies intended to demonstrate that the CRISPR/cas tool can be expressed or present in the zygote before the elimination of the haploid inducer genome, and carry the transgene necessary for editing the non-transgenic haploid genome of the elite line. Further studies of the mechanisms of the CRISPR/cas system transfer through haploid inducer lines will serve for improving them and expanding the possibility of their use as an effective tool for editing the genome of maize and other agricultural crops. The fact that some maternal haploid plants were modified with the help of a haploid inducer suggests that the sperm fuses with the egg and delivers the CRISPR/ cas system there, but the subsequent elimination of male chromosomes leads to the formation of a haploid plant with the edited genome (Kelliher et al., 2019) using the IMGE approach (Wang et al., 2019). The method developed by the authors makes it possible to obtain doubled haploid lines with the genome edited regarding the target genes during one reproduction cycle

It is also possible to obtain haploids through interspecific crossing with subsequent elimination of haploid inducer chromosomes. In barley breeding, the method of chromosomes selective elimination is widely used to obtain haploids when crossing H. vulgare L. × H. bulbosum L. (the ‘bulbosum’ method). For the first time this interspecific hybrid was obtained back in 1934. It was later established that the appearance of maternal-type haploid plants resulted not from parthenogenesis, but from the selective elimination of H. bulbosum chromosomes (2n = 2x = 14) in the alien cytoplasm of H. vulgare (2n = 2x = 14) within 9 days from the date of crossing (Gernand et al., 2006). As a result of this process, the wild-type chromosomes are eliminated and a haploid embryo is formed, while the endosperm remains underdeveloped. Therefore, 12–14 days after pollination the embryo should be transferred to the in vitro culture, and the resulting haploid regenerated plants should be colchicine- treated for double haploidization. The studies by A.P. Ermishin (Ermishin, Voronkova, 2015) described a method for obtaining dihaploids of cultivated potato Solanum tuberosum L. (2n = 2x = 24) by using a primitive cultivated potato species S. phureja (2n = 2x = 24) as a haploproducer. The method is based on the phenomenon of pseudogamy, in which both sperms of a pollen grain of a diploid species (n = x = 12) fuse with the central nucleus of the S. tuberosum embryo sac (2n = 4x = 48), which leads to the formation of a hexaploid endosperm. In this case, the unfertilized egg (n = 2x = 24) begins to differentiate; the forming viable seed combines a hexaploid endosperm with a diploid embryo (Ermishin, Voronkova, 2015).

A rather original use of the haploid induction method in breeding was found in the propagation of the dioecious asparagus (Asparagus officinalis) to increase the proportion of genotypes with low fiber content in the stem in the offspring. To this end, female (XX) and male (XY) genotypes were crossed, and the progeny showed a segregation of female fibrous and male low-fiber genotypes in the 1:1 ratio. Haploid induction in male genotypes followed by doubled haploidization of haploid genotypes results in doubled haploid super-male (YY) and female (XX) plants. The use of super-male genotypes as pollinators of female genotype makes it possible to obtain all-male (XY) plants in the F1 progeny (Bhojwani, Razdan, 1996). Haploidy is also used in a similar way to obtain same-sex genotypes for papaya (Carica papaya), but with a difference that these are female plants that are of commercial value. In this case, the anther culture method is used to obtain doubled haploid lines, which greatly facilitates the production of female pure lines (Rimberia et al., 2006).

In the doubled haploid wheat (Triticum aestivum L.) breeding, pollen from other cereal crops, such as maize, sorghum, teosinte, ornamental millet, and wild barley H. bulbosum, is used as a pollen source, however the most effective haploid inducer for wheat and oats (Avena sativa L.) remains maize pollen (Laurie, Bennett, 1988; Dziurka et al., 2022). Cytological studies have shown that maize pollen successfully germinates on the stigma of wheat, reaches the embryo sac, in which the wheat egg is fertilized by sperm from the maize pollen grain, and a zygote containing 21 wheat chromosomes and 10 maize chromosomes is formed (Laurie, 1989). The resulting hybrid zygote is karyotypically unstable, since maize chromosomes do not get attached to the division spindle and are eliminated. This process provided a basis for the original method developed by Syngenta, which used it to edit the wheat genome. To this end, a maize line NP2222 was transformed with a vector expressing Cas9 and gRNA targeting putative wheat orthologs GRASSY TILLER1 TaGT1-4A, TaGT1-4B, and TaGT1-4D. As a result, two of the 292 CMS haploids were edited, and the sequencing of the target genes showed that the JSWER30A22 gene had a 97 bp deletion in TaGT1-4B (Kelliher et al., 2019). This success evidences that the Hiedit editing method can be applied in the future to wheat for simultaneously obtaining haploid embryos with an already edited genome, which, in turn, will significantly accelerate breeding for wheat improvement (Kelliher et al., 2019).

## Conclusion

The development of applied genetics and breeding technologies opens new areas of haploid induction technique application using maize haploid inducer lines. The range of objects for the study and practical application of haploid inducers is expanding, involving not only representatives of the genus Zea, which includes its own six species, but also interspecific crosses with representatives of the genera Triticum and Avena. The first haploid inducer lines served as sources for creating many more effective haploid inducer lines due to the inclusion of various mutations that enhance the haploid induction frequency and marker properties due to the introduction of genes for anthocyanin pigmentation of grain and plants, as well as genes that control the high content of some biochemical components in the embryo. The use of maize haploid inducers for expanding the genetic polymorphism of the initial breeding material has allowed researchers and breeders to develop new technologies and methods for obtaining doubled haploid lines not only from diploid varieties, populations and synthetics, but also from polyploid maize genotypes and its wild tetraploid relatives. This method significantly accelerated hybrid maize breeding by creating new haploid inducer lines from sterile cytoplasm of S- and M-type CMS to create sterile doubled haploid maize lines. The improvement of the CRISPR/ cas method using haploid inducer lines opens up great prospects in editing the maize genome, when it becomes possible to combine two elements in one breeding operation, namely genome editing by CRISPR/cas and obtaining a completely homozygous doubled haploid line carrying these genetic changes. The research in the field of maize haploid inducer breeding has long overgrown the boundaries of application in hybrid breeding for obtaining doubled haploid lines, and every year witnesses an improvement and expansion of the area of application in applied genetics and breeding.

## Conflict of interest

The authors declare no conflict of interest.
